# Assessing structural and functional responses of murine hearts to acute and sustained β-adrenergic stimulation in vivo

**DOI:** 10.1016/j.vascn.2016.01.007

**Published:** 2016

**Authors:** Sarah-Lena Puhl, Kate L. Weeks, Antonella Ranieri, Metin Avkiran

**Affiliations:** Cardiovascular Division, King's College London British Heart Foundation Centre of Research Excellence, The Rayne Institute, St Thomas' Hospital, London SE1 7EH, United Kingdom

**Keywords:** AW, anterior wall, β-AR, β-adrenoceptor, d, end-diastolic, DOB, dobutamine, EF, ejection fraction, FS, fractional shortening, HW, heart weight, i.p., intraperitoneal, ISO, isoprenaline, IVS, interventricular septum, LV, left ventricular, LVAW, left ventricular anterior wall, LVID, left ventricular internal diameter, LVPW, left ventricular posterior wall, s, end-systolic, s.c, subcutaneous, TL, tibia length, vol, volume, Isoprenaline, Sustained β-adrenoceptor stimulation, Acute β-adrenoceptor responsiveness, Dobutamine, Methods

## Abstract

**Introduction:**

Given the importance of β-adrenoceptor signalling in regulating cardiac structure and function, robust protocols are required to assess potential alterations in such regulation in murine models in vivo*.*

**Methods:**

Echocardiography was performed in naïve and stressed (isoprenaline; 30 μg/g/day s.c. for up to 14 days) mice, in the absence or presence of acute β-adrenergic stimulation (dobutamine 0.75 μg/g, i.p.). Controls received saline infusion and/or injection. Hearts were additionally analysed gravimetrically, histologically and biochemically.

**Results:**

In naïve mice, acute β-adrenoceptor stimulation with dobutamine increased heart rate, left ventricular (LV) fractional shortening (LVFS), ejection fraction (LVEF) and wall thickness and decreased LV diameter (p < 0.05). In stressed mice, dobutamine failed to induce further inotropic and chronotropic responses. Furthermore, following dobutamine injection, these mice exhibited lower LVEF and LVFS at identical heart rates, relative to corresponding controls. Sustained isoprenaline infusion induced LV hypertrophy (increased heart weight, heart weight/body weight ratio, heart weight/tibia length ratio and LV wall thickness (p < 0.05)) by 3 days, with little further change at 14 days. In contrast, increases in LVEF and LVFS were seen only at 14 days (p < 0.05).

**Discussion:**

We describe protocols for and illustrative data from the assessment of murine cardiac responses to acute and sustained β-adrenergic stimulation in vivo, which would be of value in determining the impact of genetic or pharmacological interventions on such responses. Additionally, our data indicate that acute dobutamine stimulation unmasks early signs of LV dysfunction in the remodelled heart, even at a stage when basal function is enhanced.

## 1 Introduction

Regulation of cardiac function by the sympathetic nervous system is achieved primarily through the stimulation of cardiac β_1_-adrenergic receptors (β_1_-adrenoceptors, β_1_-AR), with acute stimulation inducing rapid and pronounced chronotropic, inotropic and lusitropic effects. Additionally, sustained β_1_-adrenoceptor stimulation induces cardiac hypertrophy and remodelling, which may be physiological or pathological in nature depending on the magnitude and duration of stimulation (Booysen et al., 2012, Kitagawa et al., 2004, Webb et al., 2010). Such effects of β_1_-adrenoceptor stimulation are achieved principally through the activation of cAMP-dependent protein kinase (protein kinase A) isoforms, although activation of other cAMP effectors, such as members of the exchange protein directly activated by cAMP (Epac) family, may also contribute in some cases.

In view of the importance of β_1_-adrenoceptor signalling pathways in regulating cardiac structure and function, it is important to have robust methods to determine how genetic manipulations (e.g. gene knock-out, knock-in or overexpression), pharmacological interventions (e.g. putative therapeutic drugs) and cardiac or systemic disease states (e.g. myocardial ischaemia, hypertension, diabetes) in mouse models may impact on such regulation. In this paper, we describe methods for the in vivo and ex vivo assessment of the effects of both acute and sustained β_1_-adrenoceptor stimulation on cardiac structure and function in wild-type mice, which may be readily adapted for use in conjunction with such settings.

## 2 Materials and methods

### 2.1 Study population

Thirty-four male C57/Bl6J mice (8–10 weeks of age, from Charles River UK Ltd., Kent, UK) were included in this study and were housed in groups of 4 in pathogen-free, individually ventilated cages with a 12-h light/12-h dark regime. Normal mouse chow containing 0.9% NaCl and water was provided ad libitum. The study was approved by the local Ethics Review Board and animal handling and experiments were performed according to the Home Office regulations, as detailed in the Home Office Guidance on the Operation of the Animals (Scientific Procedures) Act 1986, HMSO (London) and the Guide for Care and Use of Laboratory Animals by the US National Institute of Health (NIH Publication 8th edition).

### 2.2 Study design

Three different in vivo protocols were performed.

#### 2.2.1 Protocol 1: acute β-adrenergic stimulation

**Fig. 1:**
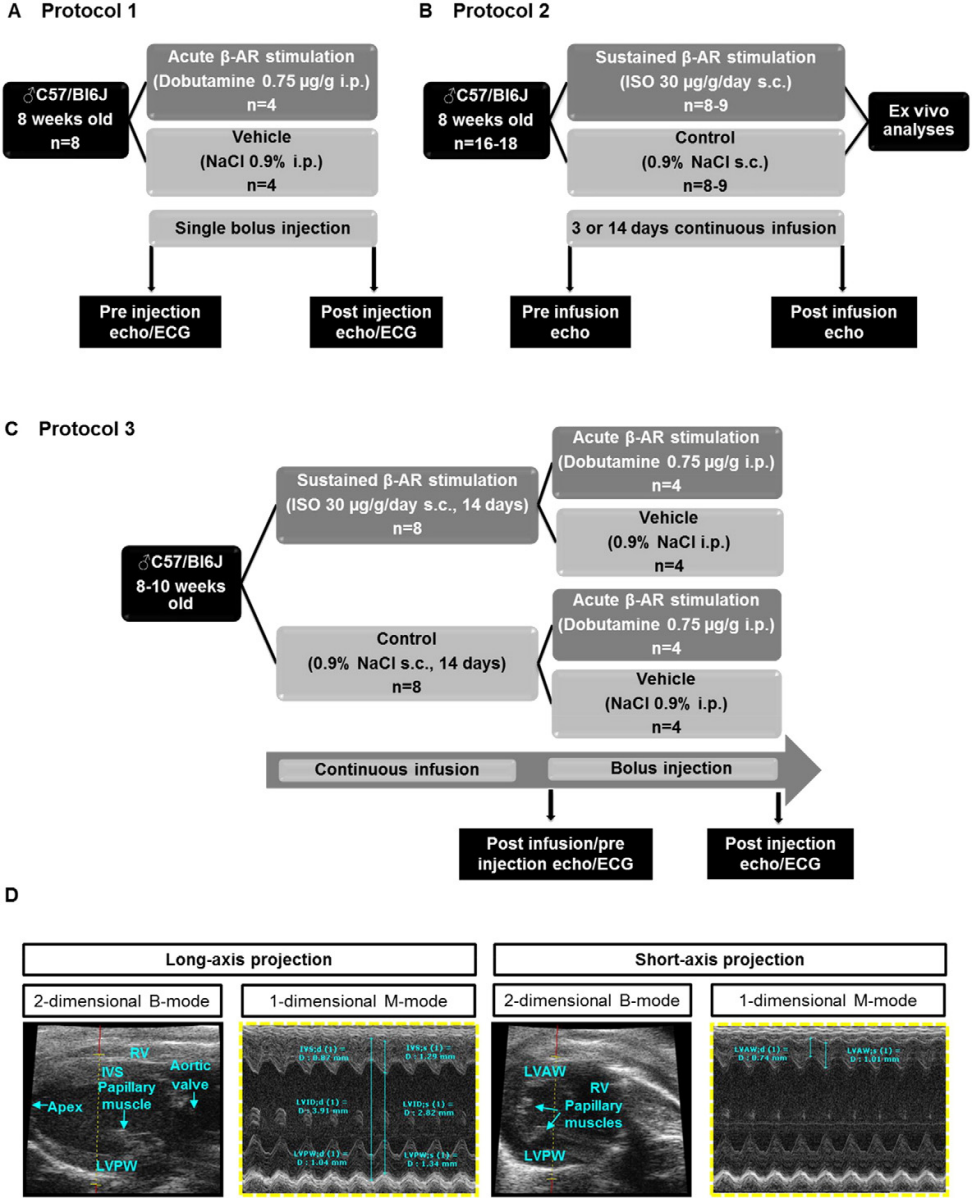
Study population and design. Experimental protocols for assessment of cardiac and other responses to (**A**) acute β-adrenoceptor stimulation with DOB, (**B**) sustained β-adrenoceptor stimulation with ISO and (**C**) acute β-adrenoceptor stimulation with DOB following sustained β-adrenoceptor stimulation with ISO. (**D**) For echocardiographic analyses, 2-dimensional (B-Mode) images were recorded in parasternal long- (left panel) and short-axis (right panel) projections with guided one-dimensional M-mode recordings at the mid-ventricular level, apical of the posteriomedial papillary muscle in both views. Standard measurements of inter-ventricular septum (IVS), left ventricular internal diameter (LVID) and left ventricular posterior wall (LVPW) were performed in systole and diastole in parasternal long-axis projection. Left ventricular (LV) cavity size and wall thickness were measured during at least three beats from this projection and averaged. Left ventricular anterior wall (LVAW) thickness was determined during at least three beats in systole and diastole in short-axis projection.

As illustrated in Fig. 1A, mice were randomly assigned to receive a single bolus intraperitoneal (i.p.) injection of dobutamine (DOB, 0.75 μg/g, n = 4) or vehicle (0.9% NaCl, n = 4) at an identical volume. Echocardiographic and ECG recordings were obtained before and after the single bolus injection.

#### 2.2.2 Protocol 2: sustained β-adrenergic stimulation

As illustrated in Fig. 1B, mice were randomly assigned to receive a continuous subcutaneous infusion of isoprenaline (ISO, 30 μg/g/day) or vehicle (0.9% NaCl) at an identical infusion rate (0.25 μl/h) for 3 (n = 9) or 14 (n = 8) days. Echocardiographic recordings were obtained before starting and at the end of each infusion period. Additionally, ex vivo gravimetric and histological analyses were performed on hearts excised after the 14d infusion period.

#### 2.2.3 Protocol 3: acute β-adrenergic stimulation after sustained β-adrenergic stimulation

As illustrated in Fig. 1C, mice were randomly assigned to receive a continuous subcutaneous infusion of isoprenaline (30 μg/g/day, n = 8) or vehicle (0.9% NaCl; n = 8) at an identical infusion rate (0.25 μl/h) for 14 days. At the end of the infusion period, mice from each group were randomly assigned to receive a single bolus i.p. injection of DOB (75 μg/g, n = 4) or vehicle (0.9% NaCl, n = 4) at an identical volume. Echocardiographic and ECG recordings were obtained before and after the single bolus injection.

### 2.3 Echocardiography

All imaging studies were performed using the VisualSonicsVevo® 770 imaging system (Scanhead: RMV707B, 15–45 MHz, cardiac mouse). Anaesthesia was induced for one minute in an induction chamber (3% isoflurane mixed with 97% O_2_ at a flow rate of 1 L/min). After placing the mouse in a supine position atop a pad with embedded ECG electrodes, anaesthesia was maintained via inhalation of 1.5–2% isoflurane and 98–98.5% O_2_ at a flow rate of 1 L/min using a nose mask. The ECG signal was monitored throughout the procedure. After immobilising the mouse on the echocardiography stage with tape, chest hair was removed with hair removal cream and a layer of preheated ultrasound gel was applied to the chest. Body temperature was monitored throughout the whole procedure by an inserted rectal probe and maintained within a narrow range (37.0 °C ± 1.5 °C) via the heated platform and a heat lamp.

Two-dimensional (B-Mode) images were recorded in parasternal long- and short-axis projections with guided one-dimensional M-mode recordings at the mid-ventricular level, apical of the papillary muscle in both views (Fig. 1D). Standard measurements of inter-ventricular septum (IVS), left ventricular internal diameter (LVID) and left ventricular posterior wall (LVPW) were performed in systole and diastole in parasternal long-axis projection. Left ventricular (LV) cavity size and wall thickness were measured during at least three beats from this projection and averaged. Left ventricular anterior wall (LVAW) thickness was determined during at least three beats in systole and diastole in short-axis projection (Fig. 1D). LV volume [μl] LVvol;d [7.0/(2.4 + LVID;d)] * LVID;d^3^, LVvol;s [7.0/(2.4 + LVID;s)] * LVID;s^3^, LV fractional shortening (FS) [%] [(LVID;d - LVID;s)/LVvol;d] * 100 and LV ejection fraction (EF) [%] [(LVvol;d - LVvol;s)/LVvol;d] * 100 were calculated from M-mode measurements. ECG and respiration gating were used to suppress movement artefacts.

#### 2.3.1 Dobutamine-stress-test

Dobutamine hydrochloride powder (Sigma-Aldrich, product number D 0676) was dissolved in ddH_2_O by gently heating at 37 °C for 15–20 min and vortexing to produce a 10 mg/ml DOB stock solution. DOB working solution (0.1 μg/μl) was prepared by 1:10 dilution of the stock dilution in sterile 0.9% NaCl. Mice were characterised via baseline echocardiography and ECG, before being randomly assigned to receive an intraperitoneal (i.p.) single bolus injection of DOB at a dose of 0.75 μg/g body weight or an equivalent volume (7.5 μl/g) of vehicle (0.9% NaCl). The DOB dose (0.75 μg/g i.p.) was selected on the basis of preliminary experiments using 3 different doses (0.375 μg/g, 0.75 μg/g or 1.5 μg/g i.p.), which revealed that 0.75 μg/g was sufficient to trigger a pronounced cardiac response in a separate cohort of adult C57/Bl6 male mice (data not shown). Echocardiographic B- and M-Mode scans in long- and short-axis projections and ECG recordings were repeated immediately after the single bolus injection and then periodically during 10 min, until the peak heart rate response was reached and heart rate began to decline again.

### 2.4 Osmotic mini-pump implantation

After an acclimatisation period of 7 days, mice were subjected to subcutaneous implantation of osmotic mini-pumps (ALZET model 1002, Durect Corporation, Cupertino, California, USA; supplied by Charles River UK Ltd., Kent, UK). After an incision was made on the back, slightly posterior to the scapulae, a distal subcutaneous pocket was created and the pump was located posterior to the right flank. ISO (DL-Isoproterenol hydrochloride, Sigma, St. Louis, MO, USA) was dissolved in 0.9% NaCl at a concentration calculated to deliver 30 μg/g/day for up to 15 days at an infusion rate of 0.25 μl/h. Pumps were filled with either the ISO solution or 0.9% NaCl (vehicle) and activated according to the manufacturer's instructions. During the surgical intervention, mice were anesthetised with 3% isoflurane mixed with 97% O_2_ (flow rate 1 L/min) and the mini-pumps were implanted via a 0.5 cm interscapular incision under sterile surgical conditions. The wound was closed with an interrupted suture using a 6–0 silk thread.

### 2.5 Tissue sampling and processing

At the endpoint of each experiment, mice were anaesthetised with 4% isoflurane and subsequently euthanized by cervical dislocation. Following left thoracotomy, the heart was explanted, washed, weighed, the atria were removed and the ventricular myocardium was split for biochemical and histological analyses. LV tissue for biochemistry was immediately frozen in liquid N_2_ and stored at − 80 °C. Midventricular slices for histological analyses were fixed in 4% formaldehyde within one minute after explantation of the heart and stored at 4 °C. To assess tissue congestion, wet and dry weight of lungs, liver, spleen and the left kidney were determined.

#### 2.5.1 Phosphate affinity SDS/PAGE and immunoblot analysis

For protein analysis of whole cell lysates, LV tissue was homogenised and lysed in EDTA-free standard lysis buffer. Samples were resolved by SDS-PAGE (10% acrylamide) either in standard format or containing the phosphate affinity reagent PhosTag (40 μmol/L), as described previously (Candasamy et al., 2014, Kinoshita et al., 2009). Prior to transfer of proteins to PVDF membranes, gels were incubated in transfer buffer containing 1 mmol/L EDTA for 15 min followed by a 15-min incubation in transfer buffer alone for 15 min. Acrylamide-pendant PhosTag was purchased from Wako Pure Chemical Industries Ltd. (Osaka, Japan), cTnI primary antibody was purchased from Cell Signaling (Beverly, MA), secondary antibody and enhanced chemiluminescence reagents were purchased from GE Healthcare (Buckinghamshire, UK). Expression of bis-, mono- and un-phosphorylated Troponin I was quantified using a calibrated densitometer (BioRad GS800).

#### 2.5.2 Histology

Myocardial collagen content (fibrosis) and myocyte cross-sectional area were assessed following staining with picrosirius red or haematoxylin and eosin (H&E), respectively, in transverse midventricular tissue slices (5 μm) of formaldehyde-fixed and paraffin-embedded murine heart tissue, as described previously (Puhl, Muller, et al., 2015).

#### 2.5.3 Data acquisition and statistical analyses

Data acquisition was performed in a blinded manner. Continuous data are presented as mean ± SE. To determine differences between two groups an unpaired student's t-test was applied. Differences between four groups were determined by two-way ANOVA (group comparison involving 2 independent variables, each one with 2 conditions), followed by Tukey's post-hoc test. All calculated p-values are two-sided. Differences with a p < 0.05 were considered to be statistically significant. All analyses were performed with Microsoft Excel 2007 and Graph Pad Prism 6.0 software.

## 3 Results

### 3.1 Acute β-AR stimulation with dobutamine

**Fig. 2:**
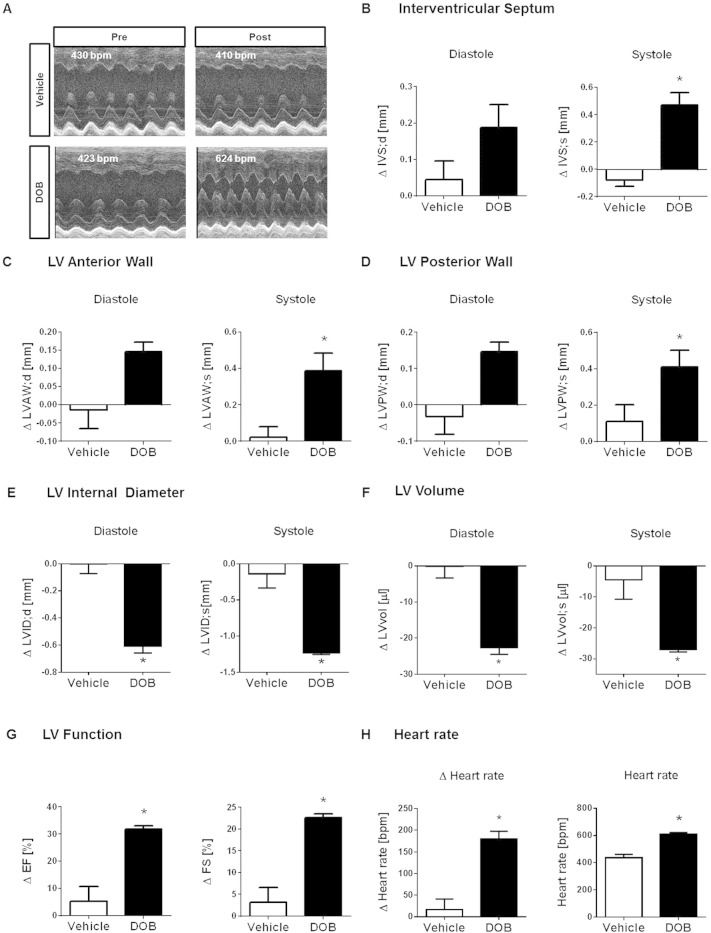
Acute β-adrenergic response assessed via echocardiography**.** (A) Representative 1-dimensional echocardiographic long-axis projections of pre (left panel) and post (right panel) i.p. single bolus vehicle (upper row) or DOB injection (lower row) and corresponding average heart rates. Changes induced by acute i.p. DOB injection in male wildtype mice in regional dimension of (**B**) IVS, (**C**) LVAW and (**D**) LVPW, (**E**) LVID and (**F**) LV volume in end-diastole and end-systole and in (**G**) LV performance, as assessed by EF, FS and (**H**) heart rate (displayed as delta and total values). Vehicle-injected mice served as control. Animal numbers per treatment n = 4; values are shown as mean ± SE, *p < 0.05 vs. vehicle; unpaired student's t-test.

Echocardiographic scans and ECG recordings were performed before (referred to as “pre injection echo/ECG”) and immediately after the single bolus DOB injection and then repeated every 2–3 min until the peak heart rate response was reached (referred to as “post injection echo/ECG”). Vehicle-injected mice were scanned in an identical manner. Fig. 2A shows representative 1-dimensional M-Mode scans obtained from 2 individual mice pre- and post-injection of a single bolus of vehicle or DOB. Pre-injection cardiac phenotypes did not differ between the groups, as shown in Table 1. For each echocardiographic parameter, the change (∆) between pre- and post-injection values was calculated in individual vehicle- and DOB-injected mice and averaged per treatment group. Compared to vehicle administration, DOB injection evoked a positive inotropic response that was reflected by pronounced regional thickening of the contracted IVS, LVAW and LVPW (Fig. 2B–D). As a consequence, end-diastolic and end-systolic LVID and LV volume decreased during the acute β-AR response (Fig. 2E–F). Based on the end-diastolic and end-systolic LVID measurements, functional parameters such as EF and FS were calculated (as described in the Methods section). There was a robust increase in both parameters following DOB injection but not vehicle injection, confirming a pronounced increase in contractility following the β-adrenergic stress stimulus (Fig. 2G). As expected, the DOB-induced inotropic response was accompanied by a robust transient increase in heart rate of approximately 200 bpm (Fig. 2H).

### 3.2 Sustained β-AR stimulation with isoprenaline

**Fig. 3:**
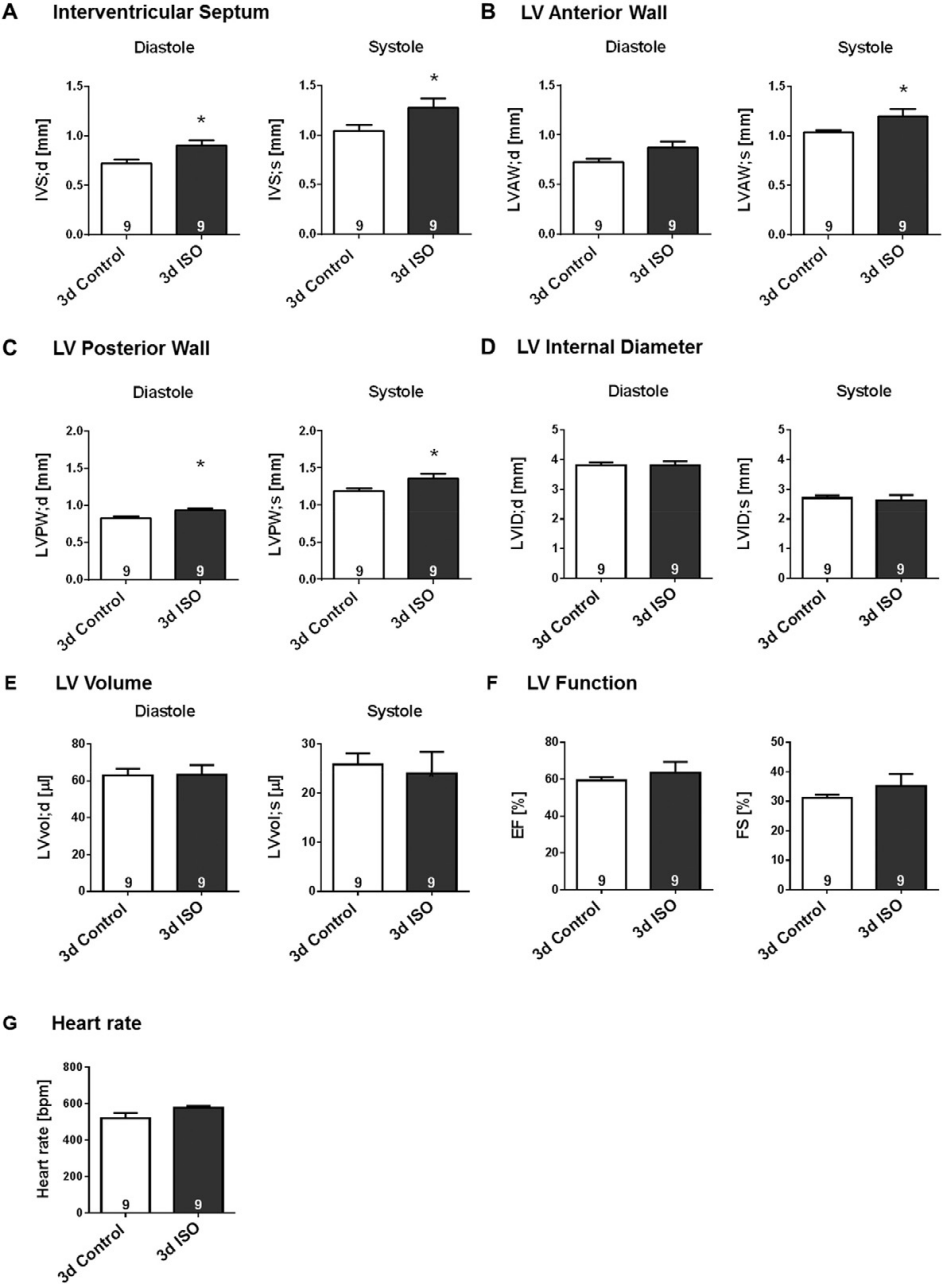
Echocardiographic evidence for moderate LV hypertrophy induced by prolonged (3-day) β-AR stimulation**.** Effect of permanent 3-days s.c. ISO infusion in male wildtype mice on regional dimension of (**A**) IVS, (**B**) LVAW and (**C**) LVPW, (**D**) LVID and (**E**) LV volume in end-diastole and end-systole and on (**F**) LV performance, as assessed by EF, FS and (**G**) heart rate. Saline-infused mice served as control. Animal numbers per treatment n = 9; values are shown as mean ± SE, *p < 0.05 vs. Control; unpaired student's t-test.

**Fig. 4:**
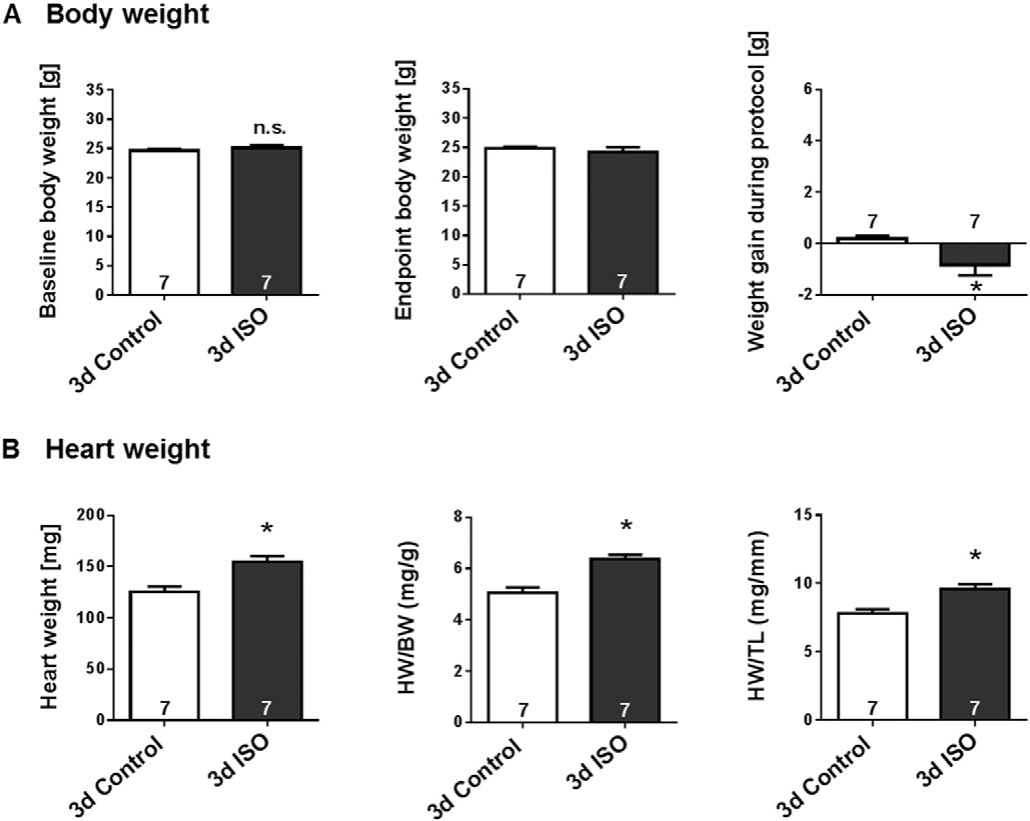
Gravimetric evidence for moderate LV hypertrophy induced by prolonged (3-day) β-AR stimulation. Effect of 3-days s.c. ISO infusion in male wildtype mice on (**A**) body weight and (**B**) heart weight as assessed by gravimetric analyses. Saline-infused mice served as control. Animal numbers per treatment n = 7; values are shown as mean ± SE, *p < 0.05 vs. Control; unpaired student's t-test.

Modest LV hypertrophy could be detected after only 3-days continuous ISO-infusion, as reflected by significant thickening of IVS, LVAW and LVPW throughout the cardiac cycle (Fig. 3A–C, p < 0.05). These structural changes were not accompanied by a decrease in LV cavity sizes (Fig. 3D–E). Mice infused with ISO for 3 days showed a trend towards increased cardiac function and heart rate, but the difference from control animals infused with saline was not statistically significant (Fig. 3F–G). Gravimetric analyses (Fig. 4A**)** revealed a small weight loss in ISO-infused mice, in contrast to a small weight gain in saline-infused mice, during the 3-day treatment period. This was accompanied by a greater lung weight without evidence of congestion (Table 2). In agreement with the echocardiographic evidence of increased LV wall thickness, ISO-infused mice exhibited significantly greater heart weight, heart weight/body weight ratio (HW/BW) and heart weight/tibia length ratio (HW/TL) relative to the saline-infused mice (Fig. 4B; p < 0.05).

**Fig. 5:**
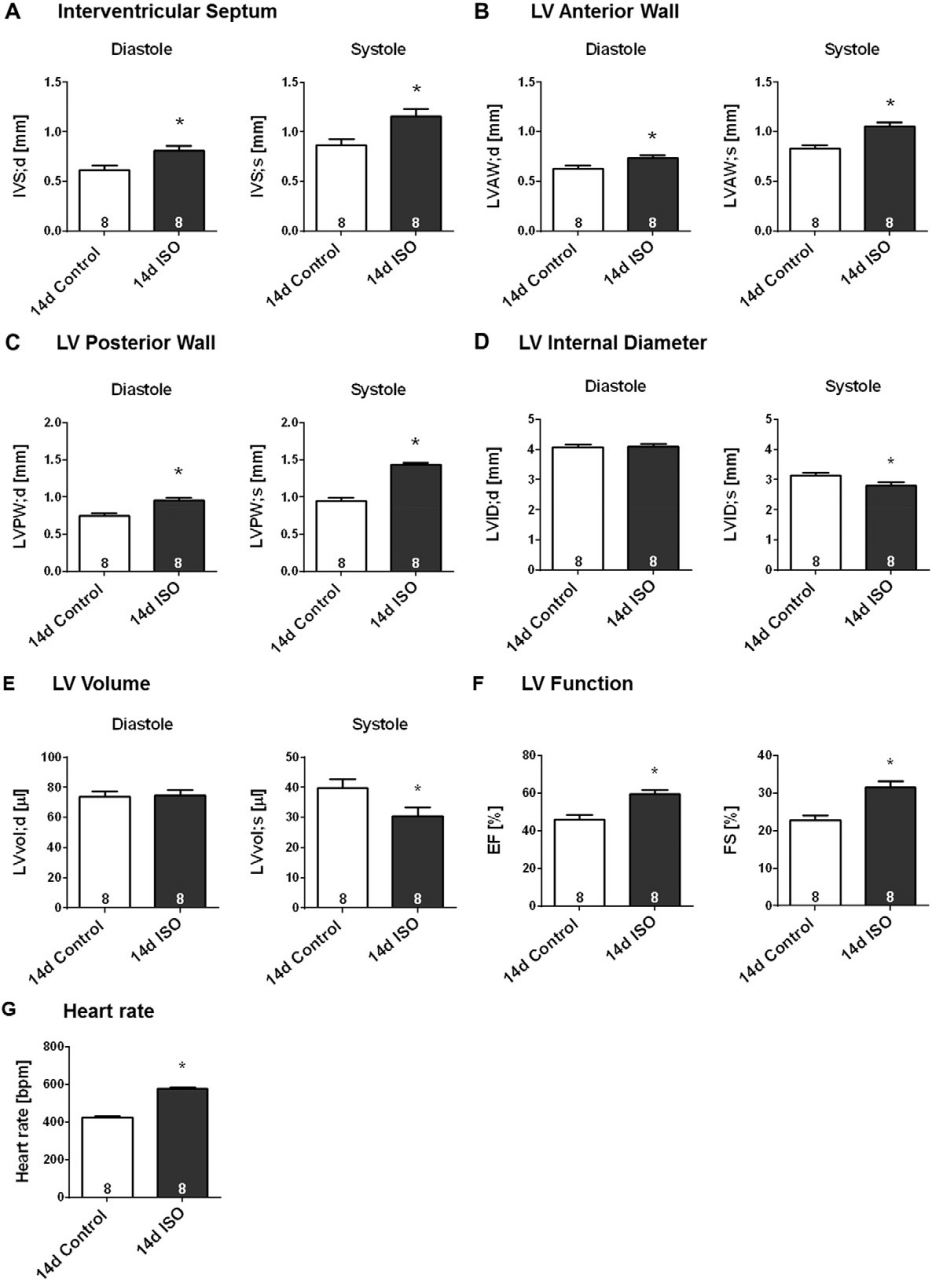
Echocardiographic evidence for LV hypertrophy induced by sustained (14-day) β-AR stimulation. Effect of sustained 14-days s.c. ISO infusion in male wildtype mice on regional dimension of (**A**) IVS, (**B**) LVAW and (**C**) LVPW, (**D**) LVID and (**E**) LV volume in end-diastole and end-systole and on (**F**) LV performance, as assessed by EF, FS and (**G**) heart rate. Vehicle-infused mice served as control. Animal numbers per treatment n = 8; values are shown as mean ± SE, *p < 0.05 vs. Control; unpaired student's t-test.

**Fig. 6:**
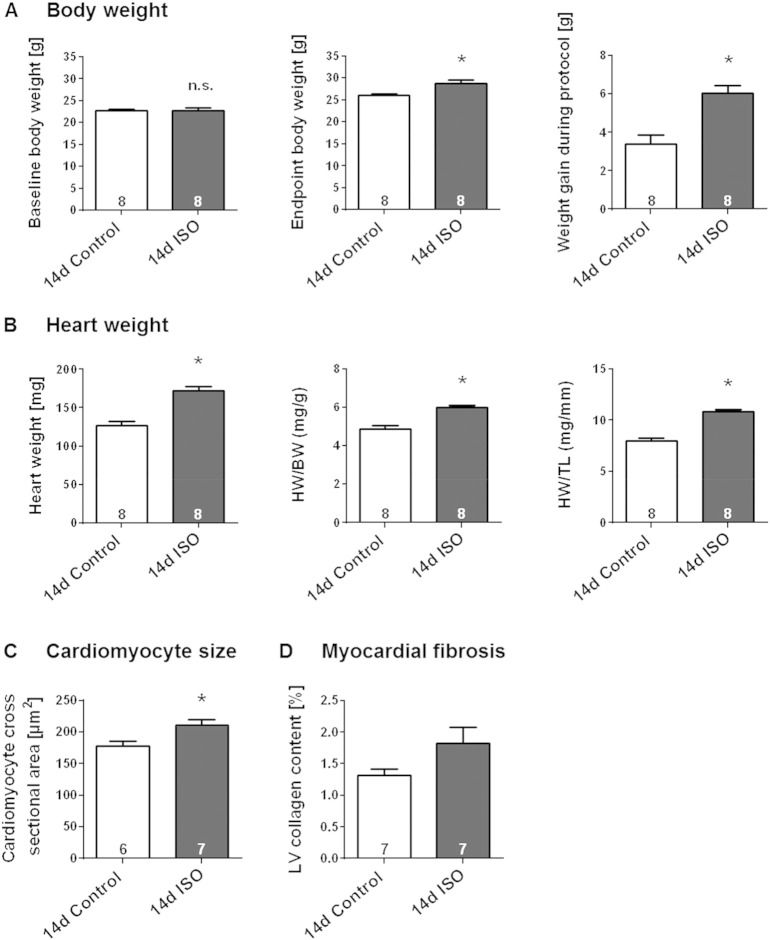
Morphological evidence for moderate LV hypertrophy induced by sustained (14-day) β-AR stimulation in the absence of myocardial fibrosis. Effect of continuous 14-days s.c. ISO infusion in male wildtype mice on (**A**) body weight, (**B**) heart weight as assessed by gravimetric analyses, (**C**) cardiomyocyte cross-sectional area and (**D**) percentage LV collagen content, as assessed via histological analyses. Saline-infused mice served as control. Animal numbers per treatment n = 8 if not indicated otherwise; values are shown as mean ± SE, *p < 0.05 vs. Control; unpaired student's t-test.

After 14-days continuous ISO-infusion, the adaptive remodelling of the murine heart could be confirmed by thickening of the IVS, LVAW and LVPW in end-diastole and end-systole (Fig. 5A–C, p < 0.05). This hypertrophic response was not accompanied by an increase in the diastolic LV cavity size, a feature of maladaptive dilatation, but rather by a significant decrease in the systolic cavity size, indicating enhanced contraction (Fig. 5D–E). Indeed, enhanced cardiac performance in the ISO group was reflected also by a significant increase in EF, FS and heart rate (Fig. 5F–G, p < 0.05). Continuous ISO infusion for 14 days provoked a gain in body weight (Fig. 6A). This was accompanied by significantly greater weights of the atria, the lungs and the liver without incidence of congestion (Table 3). In agreement with the data obtained by echocardiography, the ISO-induced hypertrophic phenotype was characterised by significant increase in heart weight, heart weight/body weight ratio (HW/BW) and heart weight/tibia length ratio (HW/TL) (Fig. 6B; p < 0.05). Consistent with these observations, ISO-infused mice exhibited a significant increase in cardiomyocyte cross-sectional area, as assessed by manually framing H&E stained LV myocytes (Fig. 6C). Determination of percentage LV collagen content in mid-ventricular tissue slices stained with Sirius red, as a measure of interstitial fibrosis, revealed no significant difference from saline-infused mice (Fig. 6D). Thus, in our model, sustained ISO infusion evokes a moderate compensated cardiac hypertrophic phenotype without inducing the hallmarks of maladaptive remodelling, such as LV dilatation and myocardial fibrosis.

### 3.3 Acute β-AR responsiveness following sustained β-AR stimulation

**Fig. 7:**
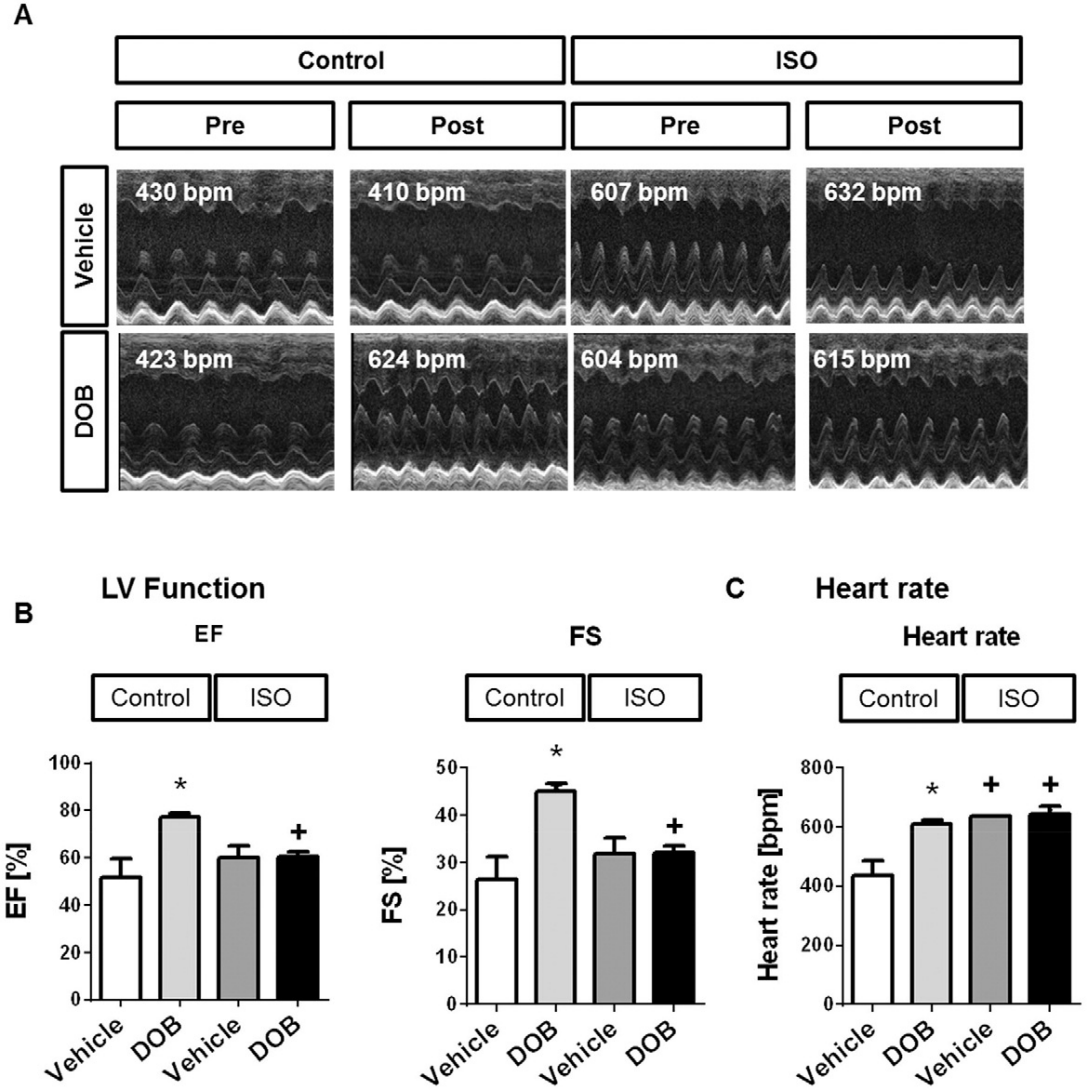
Echocardiographic evidence for loss of contractile reserve following sustained (14-day) β-AR stimulation, revealed by subsequent acute β-AR stimulation. (**A**) Representative 1-dimensional echocardiographic long-axis projections of saline-infused (Control) and ISO-infused (ISO) wild-type mice pre (left panel) and post (right panel) i.p. single bolus vehicle or DOB injection and corresponding average heart rates. Effects of acute i.p. DOB injection (DOB) in saline-infused (Control) and ISO-infused (ISO) wildtype mice on (**B**) LV function, as assessed by EF and FS, and (**C**) heart rate. Vehicle-injected mice served as control. Animal numbers per treatment n = 4; values are shown as mean ± SE, *p < 0.05 vs. corresponding vehicle, + p < 0.05 vs. corresponding control, two-way-ANOVA with Tukey's post-test.

**Fig. 8:**
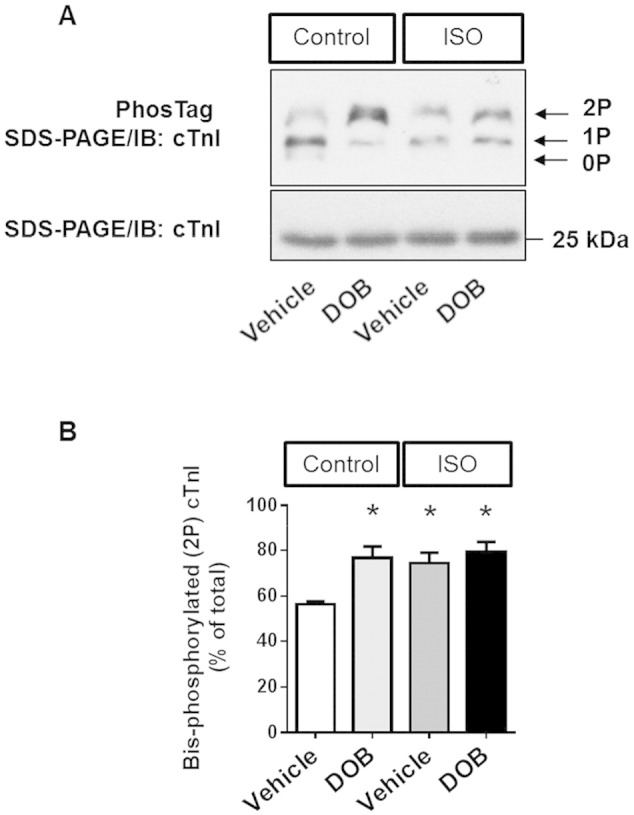
Biochemical evidence of myofilament protein phosphorylation following sustained (14-day) β-AR stimulation and subsequent acute β-AR stimulation Effects of acute i.p. DOB injection (DOB) in saline-infused (Control) and ISO-infused (ISO) wild-type mice on the relative the abundance of non-phosphorylated (0P), mono-phosphorylated (1P) and bis-phosphorylated (2P) cTnI relative to the sum of bis-, mono- and un-phosphorylated moieties. (**A**) Representative PhosTag phosphate affinity SDS-PAGE and standard SDS-PAGE immunoblots. (**B**) Quantitative data on the abundance of bis-phosphorylated (2P) cTnI, as a percentage of total cTnI (sum of the signals from all 3 cTnI phospho-moieties). Vehicle-injected mice served as control. Animal numbers per treatment n = 4; values are shown as mean ± SE, *p < 0.05 vs. vehicle-injected Control, two-way-ANOVA with Tukey's post-test.

In these experiments, DOB or vehicle was administered as a single bolus injection to mice that had just completed a 14-day period of infusion with saline or ISO. Fig. 7A shows representative 1-dimensional M-mode echocardiographic scans obtained from individual mice in each of the 4 study groups. Acute β-adrenergic stimulation with DOB produced the expected positive inotropic and chronotropic responses in mice that had received a 14-day infusion of saline (Fig. 7B–C**).** In contrast, there was no significant dobutamine response in mice that had received a 14-day infusion of ISO (Fig. 7B–C), most likely as a result of the already elevated basal LV contractility and heart rate observed in such mice (see also Fig. 5). Nevertheless, when inotropic status following acute β-adrenergic stimulation with DOB was compared between mice that had received a 14-day infusion of saline versus ISO, it was notable that the latter group showed significantly lower LVEF and LVFS values at a similarly elevated heart rate (Fig. 7B–C). These findings point towards a deficit in contractile reserve in mouse hearts that had undergone hypertrophic remodelling during sustained β-adrenergic stimulation with ISO. PhosTag SDS/PAGE-immunoblot analysis of the phosphorylation status of the myofilament protein cardiac troponin I (cTnI) showed that the abundance of bis-phosphorylated (2P) cTnI was increased significantly by DOB in the saline-infused group but not in the ISO-infused group, in which the abundance of 2P cTnI was already high basally (Fig. 8).

## 4 Discussion

In the present paper, we describe protocols for and report exemplar data from the in vivo and ex vivo assessment of murine cardiac responses to β-adrenergic stimulation in 3 distinct settings:

### 4.1 Acute β-adrenergic stimulation

For the assessment of cardiac responses to acute β-adrenergic stimulation, we used dobutamine as the pharmacological stimulus. Dobutamine is a synthetic sympathomimetic drug that targets primarily β_1_-ARs, with weaker agonist effects also at β_2_- and α_1_-ARs, and is used clinically in cardiac stress echocardiography in patients who are unable to exercise. The use of dobutamine for this application is based on its selective effects on cardiac inotropy and chronotropy with minimal direct impact on blood pressure. This minimal impact of dobutamine on blood pressure is believed to arise from the opposing effects of β_2_- versus α_1_-AR stimulation on arterial tone and the agonist's short half-life and thus transient actions. These characteristics of dobutamine have also led to its use to assess cardiac responses to acute β-adrenergic stimulation in pre-clinical studies, including in mice following either intravenous infusion (Dong et al., 2013, Wiesmann et al., 2001, Williams et al., 2001) or intraperitoneal injection (Song et al., 2010, Tyrankiewicz et al., 2013) of the agonist. In common with previous studies that utilised magnetic resonance (Tyrankiewicz et al., 2013, Wiesmann et al., 2001, Williams et al., 2001) or echocardiographic (Dong et al., 2013, Schmid et al., 2015, Song et al., 2010) imaging of cardiac function in mice, we have found that systemic dobutamine administration induces rapid, robust and transient increases in heart rate and LV contractility, the latter reflected by significantly greater thickness of the IVS, LVAW and LVPW at end-systole, as well as increased LVEF and LVFS. Following i.p. administration of the chosen 0.75 μg/g dose (which was selected on the basis of pilot experiments that also tested 0.375 and 1.5 μg/g doses), the maximum heart rate and contractility responses were achieved by approximately 5 min in each animal, and lasted for up to 15 min. This allows the rapid assessment of cardiac responses to acute β-adrenergic stimulation under light anaesthesia (1.5–2% isoflurane), during which a near-physiological basal heart rate of around 430 beats/min is maintained, and permits repeated assessments to be made, for example before and after a distinct pharmacological or surgical intervention. Based on the variability of the cardiac responses to dobutamine in our experiments in naïve animals, we can also make recommendations regarding the group sizes that may be necessary to assess the impact of these interventions, as well as genetic manipulation, on such responses. For example, on the basis of our data, an experimental group size of ≥ 4 would be necessary to detect a 20% change in the dobutamine-induced increase in LVFS, with an alpha error of 5% and a power of 80%. Our detailed description of protocols and illustrative data from the bolus administration of a rationally selected dose of dobutamine through the i.p. route (which does not require invasive vascular access) provides guidance for the ready assessment of cardiac responses to acute β_1_-AR stimulation in mice under light anaesthesia, in near-physiological conditions.

### 4.2 Sustained β-adrenergic stimulation

Cardiac responses to sustained β-adrenergic stimulation were assessed at both 3 and 14 days after the start of the continuous s.c. infusion of isoprenaline. Isoprenaline, a synthetic non-selective β-AR agonist that targets both β_1_- and β_2_-ARs, is commonly used to induce persistent adrenergic stress on the heart in rodent models, particularly when studying the pathogenesis and consequences of β-AR-mediated cardiac remodelling (Booysen et al., 2012, Kitagawa et al., 2004, Kudej et al., 1997, Webb et al., 2010). A particular advantage of isoprenaline over dobutamine for this application is the absence in the former of α_1_-AR agonist activity, which itself can induce cardiac hypertrophy (Milano et al., 1994, O'Connell et al., 2003, Puhl et al., 2015a) There are, however, sporadic reports in the literature reporting the cardiac remodelling effects also of sustained systemic administration dobutamine in rats (Buttrick, Malhotra, Factor, Geenen, & Scheuer, 1988) and mice (Anderson, Moore, & Larson, 2008). In our experiments, the s.c. infusion of isoprenaline at a dose of 30 μg/g/day induced marked cardiac hypertrophy at 14 days after the start of infusion, as reflected by significant increases in the thickness of the IVS, LVAW and LVPW at end-diastole (as well as end-systole), the HW/BW and HW/TL ratios, and cardiomyocyte size. Importantly, the phenotype that we observed seemed to reflect the development of compensatory hypertrophy, rather than maladaptive remodelling, since there was no evidence of LV dilatation or significant myocardial fibrosis, and LVEF and LVFS were significantly enhanced, at this time point. While this observation is consistent with a previous report utilising the sustained s.c. administration of isoprenaline in mice through an osmotic mini-pump (Schmid et al., 2015), other studies have noted evidence of maladaptive remodelling following persistent β-AR stimulation or over-expression (Grimm et al., 2015, Webb et al., 2010, Wiesmann et al., 2001, Yan et al., 2015). Such differences in findings may be a consequence of the use of different doses or durations of isoprenaline treatment (of note, in some studies reporting maladaptive remodelling, higher doses of isoprenaline (Tang et al., 2013, Yan et al., 2015) or longer treatment periods (Booysen et al., 2012) were used), and different mouse strains and/or ages. Interestingly, in the present study, our echocardiographic and gravimetric analyses of cardiac structure and function after only 3 days of isoprenaline infusion indicated that the development of compensatory hypertrophy occurs predominantly within this time-frame after the initiation of continuous β-adrenergic stimulation. These observations suggest that investigation of the initial triggering pathways and mechanisms underlying β-AR-mediated compensatory hypertrophy should be focused on the initial hours and days following the start of isoprenaline infusion. Once again, based on the variability of the cardiac responses to sustained isoprenaline infusion in our experiments in naïve animals, we can make recommendations regarding the group sizes that may be necessary to assess the impact of pharmacological, surgical or genetic manipulations on such responses. As an example, our data indicate that to detect a 20% change in the isoprenaline-induced increase in the HW/BW ratio at the 3-day time point, with an alpha error of 5% and a power of 80%, an experimental group size of ≥ 4 would be necessary.

### 4.3 Acute β-adrenergic stimulation after sustained β-adrenergic stimulation

To explore the potential of acute β-adrenergic stimulation to reveal any deficits in cardiac function in remodelled hearts, which may not be apparent in the basal state, we also examined the effects of a single bolus injection of dobutamine following a 14-day infusion of isoprenaline. Indeed, the application of the dobutamine stress-test revealed that a loss of contractile reserve is present even at a stage of compensatory hypertrophy that is accompanied by elevated basal cardiac function. The mechanisms underlying such a deficit cannot be determined on the basis of the present study, but may include abnormalities in excitation–contraction coupling, sarcomeric protein function, myocardial perfusion (for example, through reduced capillary density) or metabolism. Nevertheless, the desensitisation of cardiac β-ARs seems an unlikely causal factor since the heart rate of the ISO-infused animals was maintained at an elevated level basally (637 ± 1 bpm) and was comparable to that of saline-infused animals following dobutamine injection (611 ± 12 bpm). This suggestion is supported at a biochemical level by our observations on the phosphorylation status of cTnI, whose bis-phosphorylation by protein kinase A (PKA) downstream of β_1_-AR stimulation is a key mechanism regulating cardiac twitch dynamics (Pi, Kemnitz, Zhang, Kranias, & Walker, 2002). Our data show that the abundance of bis-phosphorylated (2P) cTnI was elevated in ISO-infused mice relative to saline-infused mice and was comparable to that observed in saline-infused mice following dobutamine injection. This indicates the presence of a functional β_1_-AR/PKA signalling module in the myocardium of ISO-infused mice. Of note, using magnetic resonance imaging, Wiesmann and colleagues (Wiesmann et al., 2001) have previously reported analogous findings, by showing that the acute i.p. administration of dobutamine helps unmask evidence of diastolic dysfunction in a distinct mouse model of compensatory cardiac hypertrophy without abnormalities in basal LV function (4-month-old transgenic mice with cardiac-specific overexpression of the β_1_-AR). Taken together, our findings and those of Wiesmann and colleagues (Wiesmann et al., 2001) indicate that assessment of the cardiac effects of the acute i.p. administration of dobutamine in the closed-chest mouse under light anaesthesia is a valuable method that may reveal contractile deficits arising from distinct pharmacological, surgical or genetic interventions that are not apparent under basal, unstressed conditions.

To conclude, in the present paper, we describe detailed methodology for and provide illustrative data from the assessment of the functional and structural cardiac responses to acute and sustained β-adrenergic stimulation in mice in vivo, which we hope will be helpful to cardiovascular investigators in assessing the potential impact of pharmacological, surgical or genetic interventions on such responses in pre-clinical studies.

## Funding

This work was supported by the British Heart Foundation, through a Project Grant (PG/12/48/29,638) and a Centre of Research Excellence Award (RE/13/2/30,182).

## Conflict of interest

None.

## Author contributions

Sarah-Lena Puhl

• Has designed the experiments.
• Has performed all in vivo experiments and ex vivo histological analyses.
• Has written and submitted the manuscript.

Kate L. Weeks

• Has designed the experiments.
• Has performed ex vivo cardiac hypertrophy analyses.
• Has reviewed and approved the final version of the manuscript for submission.

Antonella Ranieri

• Has performed protein phosphorylation analyses.
• Has reviewed and approved the final version of the manuscript for submission.

Metin Avkiran

• Has designed the experiments.
• Has written the manuscript.
• Has reviewed and approved the final version of the manuscript for submission.

## Figures and Tables

**Table 1:** Echocardiographic baseline characterisation before single bolus injection.

	Pre-vehicle injection (n = 4)	Pre-DOB injection (n = 4)
IVS; d [mm]	0.56 ± 0.03	0.68 ± 0.08
IVS; s [mm]	0.88 ± 0.08	0.85 ± 0.08
LVAW; d [mm]	0.63 ± 0.04	0.64 ± 0.05
LVAW; s [mm]	0.79 ± 0.04	0.87 ± 0.05
LVPW; d [mm]	0.79 ± 0.05	0.71 ± 0.05
LVPW; s [mm]	0.96 ± 0.05	0.94 ± 0.08
LVID; d [mm]	4.20 ± 0.11	3.97 ± 0.04
LVID; s [mm]	3.23 ± 0.16	3.01 ± 0.05
LVvol; d [μl]	78.92 ± 5.00	69.00 ± 1.73
LVvol; s [μl]	42.42 ± 4.85	37.46 ± 1.41
EF [%]	46.74 ± 3.58	45.55 ± 2.37
FS [%]	23.29 ± 2.11	22.38 ± 1.42
Heart rate	421 ± 7	430 ± 7

Data are mean ± SE.

**Table 2:** Effects of short-term (3d) ISO infusion on organ weights.

Organ		Weight (mg)	
Control (n = 7)	ISO (n = 7)
Atria	Wet	9.4 ± 1.0	11.4 ± 0.3
Lungs	Wet	133.4 ± 3.9	152.4 ± 3.9⁎
	Dry	32.2 ± 1.1	36.1 ± 1.2⁎
Liver	Wet	1212 ± 73	1059 ± 96
	Dry	429 ± 26	353 ± 35
Spleen	Wet	77.1 ± 2.7	95.9 ± 14.5
	Dry	19.1 ± 0.7	23.7 ± 3.4
Kidney	Wet	149.0 ± 6.2	152.0 ± 7.9
	Dry	40.2 ± 1.6	40.6 ± 1.8

Data are mean ± SE.

⁎ p < 0.05 vs. Control.

**Table 3:** Effects of sustained (14d) ISO infusion on organ weights.

Organ		Weight (mg)	
Control (n = 8)	ISO (n = 8)
Atria	Wet	8.6 ± 0.8	12.1 ± 0.7⁎
Lungs	Wet	142.2 ± 3.8	168.6 ± 3.8⁎
Dry	33.2 ± 0.8	38.6 ± 0.7⁎
Liver	Wet	1150 ± 84	1351 ± 10⁎
Dry	360 ± 10	422 ± 10⁎
Spleen	Wet	76.1 ± 3.6	78.5 ± 3.1
Dry	18.4 ± 0.9	18.7 ± 0.7
Kidney	Wet	141.7 ± 4.6	152.7 ± 4.6
Dry	36.4 ± 1.2	38.2 ± 1.0

Data are mean ± SE.

⁎ p < 0.05 vs. Control.
